# Sex Differences in Depression: Insights from Multimodal Gray Matter Morphology and Peripheral Inflammatory Factors

**DOI:** 10.3390/ijms252413412

**Published:** 2024-12-14

**Authors:** Wenjun Wang, Wenjia Liang, Chenxi Sun, Shuwei Liu

**Affiliations:** 1 Shandong Key Laboratory of Mental Disorders, Department of Anatomy and Neurobiology, Institute for Sectional Anatomy and Digital Human, School of Basic Medical Sciences, Cheeloo College of Medicine, Shandong University, Jinan 250012, China; 2 Institute of Brain and Brain-Inspired Science, Shandong University, Jinan 250012, China

**Keywords:** major depressive disorder, sex difference, inflammatory factors, IL-8, brain morphology, microstructure, gray-matter-based spatial statistics

## Abstract

Major depressive disorder (MDD) exhibits notable sex differences in prevalence and clinical and neurobiological manifestations. Though the relationship between peripheral inflammation and MDD-related brain changes is well studied, the role of sex as a modifying factor is underexplored. This study aims to assess how sex influences brain and inflammatory markers in MDD. We utilized voxel-based and surface-based morphometry to analyze gray matter (GM) structure, along with GM-based spatial statistics (GBSS) to examine GM microstructure among treatment-naive patients with depression (n = 174) and age-matched healthy controls (n = 133). We uncovered sex-by-diagnosis interactions in several limbic system structures, the frontoparietal operculum and temporal regions. Post hoc analyses revealed that male patients exhibit pronounced brain abnormalities, while no significant differences were noted in females despite their higher depressive scores. Additionally, heightened inflammation levels in MDD were observed in both sexes, with sex-specific effects on sex-specific brain phenotypes, particularly including a general negative correlation in males. Intriguingly, mediation analyses highlight the specific role of the parahippocampal gyrus (PHG) in mediating interleukin (IL)-8 and depression in men. The findings suggest that in clinical practice, it would be beneficial to prioritize sex-specific assessments and interventions for MDD. This includes recognizing the possibility that male patients may experience significant brain alterations, especially when identifying male patients who may underreport symptoms. Possible limitations encompass a small sample size and the cross-sectional design. In future research, the incorporation of longitudinal studies or diverse populations, while considering illness duration, will enhance our understanding of how inflammation interacts with brain changes in depression.

## 1. Introduction

Major depressive disorder (MDD) is a heterogeneous mental disorder that has become a serious public health problem worldwide and that has a notable sex gap in both epidemiological and non-epidemiological studies despite affecting both men and women [1]. Females are about twice as likely to be diagnosed with MDD compared to males, with this disparity emerging after puberty and persisting across different age groups [2]. Women with MDD exhibit symptoms such as weight gain, appetite disturbance, hypersomnia, and a higher likelihood of comorbid anxiety, while men often appear irritable, engage in substance use and risk-taking behaviors, and face a higher risk of suicide [3,4]. Evidence suggests that sexual dimorphism may be a risk factor for depression and that the neurobiological basis for these sex differences in MDD may be distinct [5]. A detailed description of these neurobiological underpinnings would be valuable for developing targeted treatment strategies for women and men with MDD [6].

Clinical research indicates distinct brain magnetic resonance imaging (MRI) signatures for males and females with MDD [7,8]. Researchers observed that females with MDD exhibited a significantly reduced surface area in the left ventrolateral prefrontal (vlPFC), decreased cortical volume in the right rostromedial PFC (rmPFC), and an increased local gyrification index in the left visual cortex using FreeSurfer software. Conversely, males with MDD displayed significant differences in the opposite direction [8]. Another study emphasized that the association between dorsomedial PFC (dmPFC) gray matter volume (GMV) and depression levels was significantly stronger in men than in women, based on non-clinical samples of both sexes [9]. Diffusion tensor imaging (DTI), which measures the proportion of free water within a voxel, is commonly used for characterizing white matter microstructural properties, several studies also utilize it to investigate GM alterations in various neuropsychiatric disease and cognitive disorders [10,11]. Using diffusion metrics, Lyon et al. found sex-specific differences in fiber bundles associated with MDD in the anterior limb of the internal capsule in men and the genu of corpus callosum in women [12]. Another study on depressed adolescents found that depressed females showed higher R1 values (the inverse of the T1 signal and can be used to infer tissue types such as myelin) in the left uncinate fasciculus and corpus callosum genu than healthy females, while no significant differences were found in males [13]. However, there is a paucity of research examining the microstructural properties of GM in MDD. Moreover, the current results regarding morphological studies in MDD are inconsistent, potentially due to small samples and varying ages, onset times, and treatment statuses [4], highlighting the necessity for systematic research on sex differences in GM associated with MDD.

Considerable evidence suggests a significant dialogue between peripheral inflammatory factors and the central nervous system (CNS) in individuals with MDD [14]. Notably, researchers have employed a safe and mild experimental inflammation approach via typhoid vaccination that yielded mood deterioration alongside notable alterations in brain function/structure and behavior [15]. A recent major discovery revealed that matrix metalloprotease 8 (MMP8), a member of the collagenase family that is primarily secreted by neutrophils and monocytes, is upregulated in the sera of individuals with MDD and stress-induced mice [16,17]. Moreover, peripheral MMP8 functions by passing into the brain parenchyma, leading to neurophysiological changes in the extracellular matrix (ECM) and neuronal activity, ultimately resulting in altered social behavior [18]. Additionally, a growing body of studies suggests that periphery pro-inflammatory cytokines can enter the CNS through a damaged blood–brain barrier (BBB) or the dural lymphatic system [19]. Numerous studies have examined the relationship between peripheral inflammation and brain changes in MDD [20,21]. However, it remains unclear whether there are differences in these inflammatory factors between female and male individuals with MDD and how these markers exert sex-specific influence on the structural imaging characteristics that exhibit variations between the sexes in MDD.

To bridge this gap, we initially conducted a comprehensive assessment of sex-related differences in GM structure and microstructure in 174 treatment-naive individuals with MDD and 133 age-matched healthy young adults. We employed surface-based morphometry (SBM) to capture cortical thickness, voxel-based morphometry (VBM) for whole-brain GMV analysis including subcortical structures, and data-driven GM-based spatial statistics (GBSS) to extract the microstructural properties. Following this, the plasma concentrations of MMP8, active MMP8, pro-inflammatory cytokines tumor necrosis factor (TNF) -α, interleukin (IL) -6, IL-8, and anti-inflammatory cytokine IL-10 are collected. We assessed the inflammatory levels in individuals with MDD and examined the role of inflammation in structuring the observed sex-by-diagnosis interactions in brain metrics. We identified a pivotal inflammatory marker exhibiting a significant sex-by-diagnosis interaction and analyzed whether sex-related brain structural phenotypes in MDD mediate the relationship between this marker and depressive symptoms. This study provides new insights into the relationship between brain structure, inflammation, and sex differences in depression, offering a fresh perspective on the conventional understanding of how depression manifests differently across sexes.

## 2. Results

### 2.1. Demographic and Clinical Features

The demographic and clinical characteristics are outlined in Table 1. There was no significant difference in age among the four groups. Female individuals with MDD exhibited significantly higher scores on the Hamilton Depression Rating Scale-17 (HAMD-17) and Beck Depression Inventory-II (BDI-II) scales compared to male individuals with MDD (***p* < 0.001**). They also reported a higher prevalence of self-injury behavior than male individuals (***p* = 0.03**). However, there were no statistically significant differences in suicidal ideation (*p* = 0.65), suicidal attempts (*p* = 0.79), and experience of childhood trauma (*p* = 0.76) between males and females with MDD. Moreover, a significant increase in alcohol consumption was noted among females with MDD when compared to their healthy counterparts.

**Figure 1:**
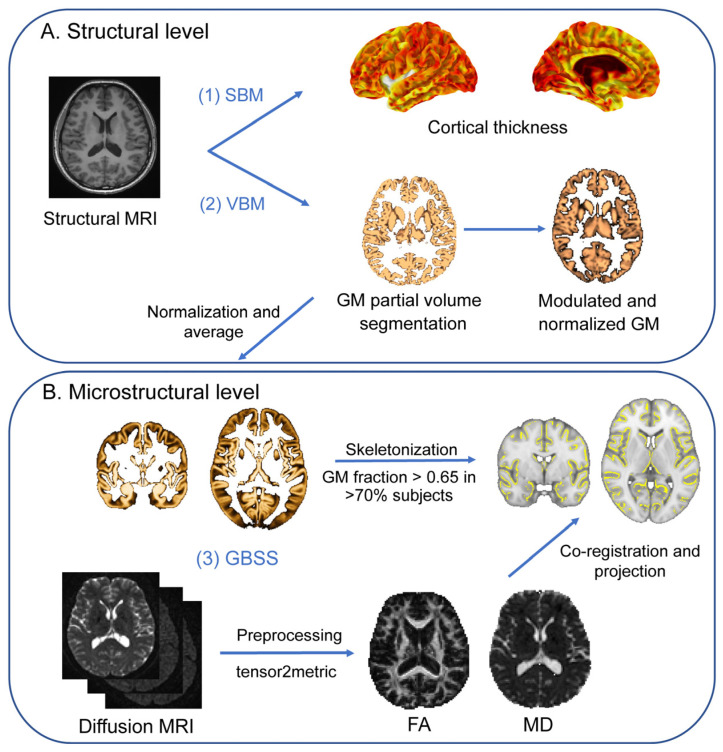
Brief pipeline for the multimodal metric of GM. (**A**) Structural-level metric of GM. (**B**) Microstructural-level metric of GM. Note: GM, gray matter; SBM, surface-based morphometry; VBM, voxel-based morphometry. GBSS, GM-based spatial statistic; MD, mean diffusivity; FA, fractional anisotropy.

Neuroimaging analyses in this study is outlined by a concise pipeline consisting of two main parts. Figure 1A illustrates a concise overview of the pipeline for the structural-level metrics of GM. Figure 1B demonstrates a concise overview of the pipeline for the microstructural-level metrics of GM.

### 2.2. Effects of MDD and Sex on GM Morphometry

**Figure 2:**
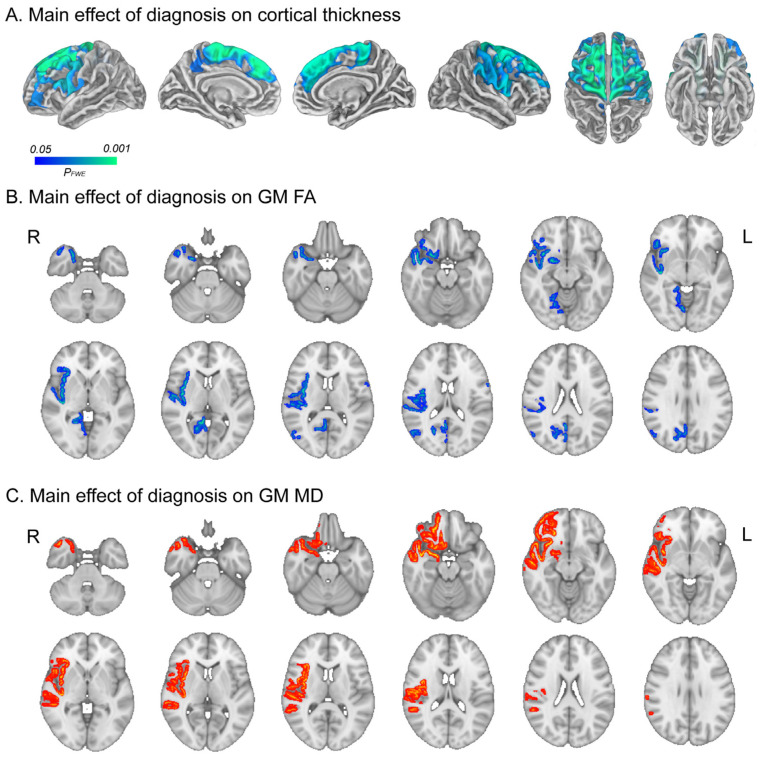
Main effects of MDD diagnosis. The main effect of MDD on cortical thickness (**A**), GM FA (**B**), and MD (**C**). Areas in blue-green indicate regions where the cortical thickness (**A**) and GM FA (**B**) are significantly lower in the MDD group compared with HCs regardless of sex. The red-yellow areas are regions where the MD (**C**) is significantly higher in the MDD group compared with HCs. All statistical significances were determined at *p* < 0.05 after applying FWE correction for multiple comparisons following TFCE while controlling for age and BMI. Note: MDD, major depressive disorder; GM, gray matter; HC, healthy control; MD, mean diffusivity; FA, fractional anisotropy; TFCE, threshold-free cluster enhancement; FEW, family-wise error rate.

The mixed-sex MDD group did not show any significant changes in the GMV compared to healthy participants after FWE correction, though they exhibited a widespread reduction in cortical thickness, particularly in the bilateral dorsal PFC and frontoparietal operculum (Figure 2A). Individuals with MDD were also found to exhibit significant alterations in the diffusion characteristics of GM, specifically a decrease in FA (Figure 2B) and an increase in MD (Figure 2C). The trend toward decreased anisotropy and higher water dispersion in individuals with MDD was observed in the right operculum, insula, temporal lobes, and limbic structures including the olfactory cortex, hippocampus, amygdala, and parahippocampal gyrus (PHG). Additionally, reduced FA was also observed in the right precuneus and posterior cingulate gyrus (PCC), while increased MD was found in the right orbitofrontal cortex (OFC) (Figure 2B).

Regarding the main effect of sex, this study revealed mutually supportive outcomes that encompass both overlapping and distinct brain regions across different modalities, indicating that females exhibit higher levels of cortical thickness (Figure S3A), relative GMV (Figure S3B), and GM FA (Figure S3C), accompanied with reduced MD (Figure S3D). Meanwhile, men exhibited higher relative GMV in the bilateral lingual gyrus and FA in the left pars opercularis compared to women (Figure S3B). These findings further validate a robust pattern of sexual dimorphism in brain structure and microstructure.

### 2.3. Interaction Effect of MDD and Sex on GM Morphometry

**Figure 3:**
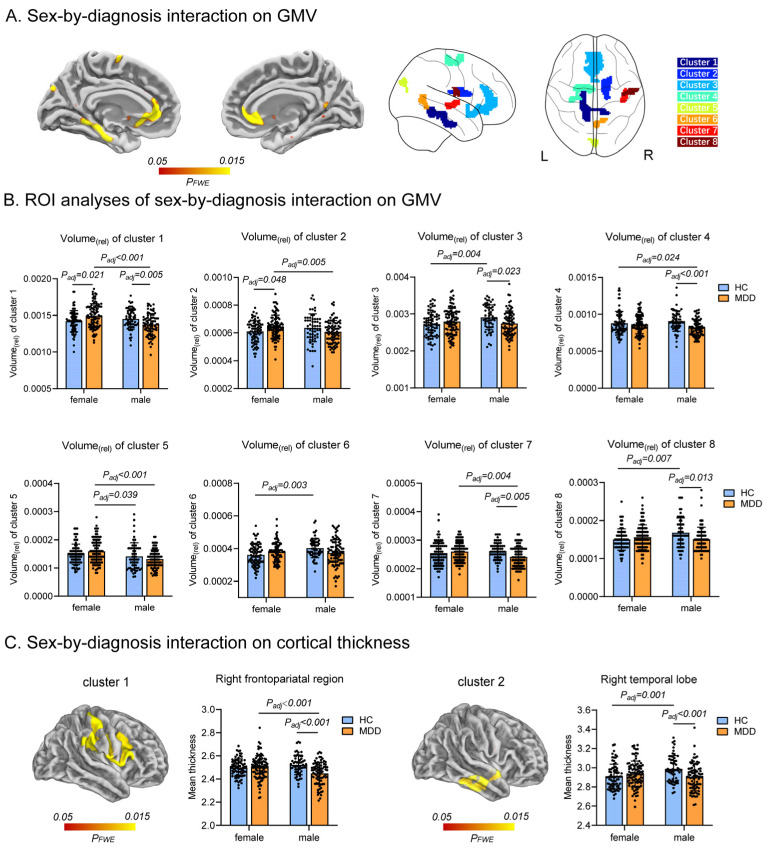
Sex-by-diagnosis interaction on GMV and cortical thickness. (**A**) The red-yellow areas indicate a significant sex-by-diagnosis interaction on GMV (*p* < 0.05 after FWE correction for multiple comparisons following TFCE). (**B**) Histograms exhibit the validation of region of interest (ROI) analysis in the original individual space for each cluster determined by the interaction of sex and diagnosis on GMV. (**C**) (**Left**): The red-yellow areas indicate a significant sex-by-diagnosis interaction on cortical thickness (*p* < 0.05 after FWE correction for multiple comparisons following TFCE). (**Right**): The histograms display ROI validation in raw space for each cluster determined by the interaction of sex and diagnosis on cortical thickness. Note: GMV, gray matter volume; Vol_(rel)_, relative volume; MDD, major depressive disorder; HC, healthy control; F, female; M, male.

Although no significant volumetric changes were observed in the main effect of MDD, we identified eight clusters exhibiting significant sex-by-diagnosis interactions, indicating that the GMV of these brain regions is influenced by both MDD and sex. These variations were found in various local brain regions, including the bilateral anterior cingulate cortex (ACC), olfactory cortex, precuneus, and calcarine cortex; left PHG, nucleus accumbens (NAc), upper part of precentral and postcentral gyri, and cuneus; right caudate nucleus, insula, superior temporal gyrus (STG), and Rolandic operculum (Figure 3A). Meanwhile, two clusters exhibiting sex differences in cortical thickness were observed in the right supramarginal, postcentral gyrus, frontoparietal operculum, and temporal regions among individuals with MDD (Figure 3C). The Supplementary Tables S2 and S3 provide detailed information regarding the brain regions that exhibit statistically significant sex differences. No significant interaction between sex and diagnosis was found in the diffusion properties of GM.

To substantiate the sex-by-diagnosis interactions observed in template space, we initially computed the relative volume and mean cortical thickness for each cluster in the native space of individual subjects. Our analysis revealed significant interactions between sex and diagnosis, thereby reinforcing our template space findings (Figure 3B,C). Moreover, the effect sizes of volumes and thicknesses of the brain regions demonstrating an interaction effect fall within the range of moderate to small magnitudes with age and body mass index (BMI) as covariates (partial η^2^ range = 0.017–0.062, see Supplementary Table S4). Specifically, we observed that males with MDD generally exhibit a significantly lower volume in most clusters compared to male controls or females with MDD.

We then replicated our initial findings regarding sex-by-diagnosis interaction on GMV in an independent dataset in order to test the extent to which the results generalize to other subject samples from other countries and scanned under different conditions. The analysis was performed on the eight GMV clusters (Figure S4), and we observed that cluster 1 (left PHG) showed significant sex-by-diagnosis interaction with a partial η^2^ effect size of 0.051 (***p* = 0.012**, Table S5).

### 2.4. Sex-Specific Effect of MDD on GM Morphometry

**Figure 4:**
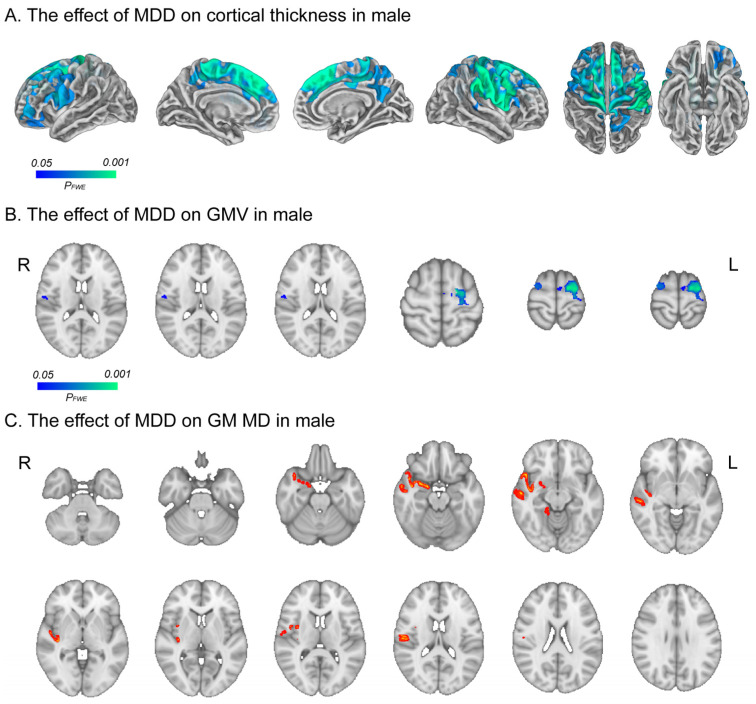
The impact of MDD on males. Areas in blue-green indicate regions where the cortical thickness (**A**) and GMV (**B**) are significantly decreased in male patients with MDD. (**C**) The effect of MDD on GM MD in males. Areas in red-yellow indicate regions where GM MD is significantly increased in male patients with MDD. All statistical significances were determined at *p* < 0.05 after applying FWE correction for multiple comparisons following TFCE while controlling for age and BMI. Note: MDD, major depressive disorder; GM, gray matter; HC, healthy control; MD, mean diffusivity; FA, fractional anisotropy; TFCE, threshold-free cluster enhancement; FEW, family-wise error rate.

Post hoc contrasts within each sex revealed that male patients exhibit pronounced structural brain abnormalities, whereas no statistically significant differences were observed for all measurements in females. In males, cortical thickness reductions were observed in regions that aligned with the main MDD effects, predominantly affecting the bilateral dlPFC, dmPFC, frontoparietal operculum, PCC, and left vmPFC (Figure 4A). Furthermore, GMV reductions in males with MDD were primarily localized to the upper segments of the bilateral precentral and postcentral gyri (Figure 4B). DTI metrics indicated an increase in MD exclusively in males with MDD, with the affected areas predominantly concentrated in the right Rolandic operculum, superior and middle temporal gyrus, insula, hippocampus, olfactory cortex, and PHG (Figure 4C).

### 2.5. Elevated Inflammation and Differential Correlations with Depressive Symptoms in Men and Women with MDD

**Figure 5:**
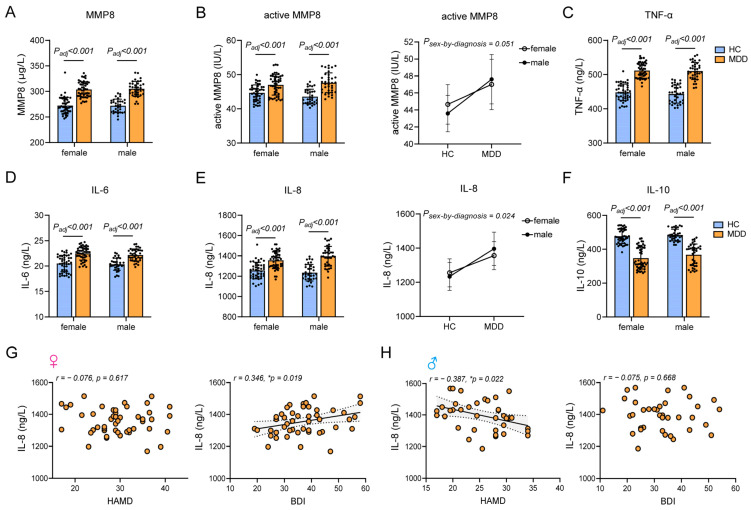
Sex differences in inflammatory factors associated with MDD. Altered plasma concentrations of MMP8 (**A**), active MMP8 (**B**), the pro-inflammatory cytokines TNF-α (**C**), IL-6 (**D**), and IL-8, and (**E**) the anti-inflammatory cytokine IL-10 (**F**) in both male and female individuals diagnosed with MDD while controlling for age and BMI. Significant sex-by-diagnosis interactions were found for IL-8 ((**E**), **right**). (**G**) Correlations between IL-8 and HAMD (**left**) or BDI (**right**) in female individuals with MDD, controlling for age and BMI. (**H**) Correlations between IL-8 and HAMD (**left**) or BDI (**right**) in male individuals with MDD, controlling for age and BMI. Note: MMP8, matrix metalloproteinase-8; TNF, tumor necrosis factor; IL, interleukin. * *p* < 0.05.

Elevated plasma concentrations of MMP8 (Figure 5A), active MMP8 (Figure 5B) and the pro-inflammatory cytokines TNF-α (Figure 5C), IL-6 (Figure 5D), and IL-8 (Figure 5E) were observed in both male and female individuals with MDD, accompanied by a decrease in the anti-inflammatory cytokine IL-10 (Figure 5F). Among these inflammatory factors, IL-8 shows a significant sex-by-diagnosis interaction when controlling for age and BMI (partial η^2^ = 0.032, ***p* = 0.024**, Figure 5E, right). Specifically, the effect size of IL-8 was larger in men (Cohen’s d = 1.920, ***p* < 0.001**, Figure 5E, left) than in women (Cohen’s d = 1.192, ***p* < 0.001**, Figure 5E, left). The findings suggest that patients with MDD exhibit elevated levels of inflammation and that male individuals with MDD demonstrate higher peripheral levels of IL-8 compared to their female counterparts. Additionally, in the analysis of the intensity of depressive symptoms, IL-8 was the only inflammatory marker found to be significantly correlated with depressive symptoms among the measured markers. Specifically, in female individuals with MDD, IL-8 showed no significant correlation with the clinician-rated HAMD but a significantly positive correlation with the self-rated BDI (Figure 5G). In contrast, in male individuals with MDD, IL-8 exhibited a significant negative correlation with the clinician-rated HAMD and no significant correlation with the self-rated BDI (Figure 5H).

### 2.6. Sexually Divergent Inflammation–Brain Correlations in MDD

**Figure 6:**
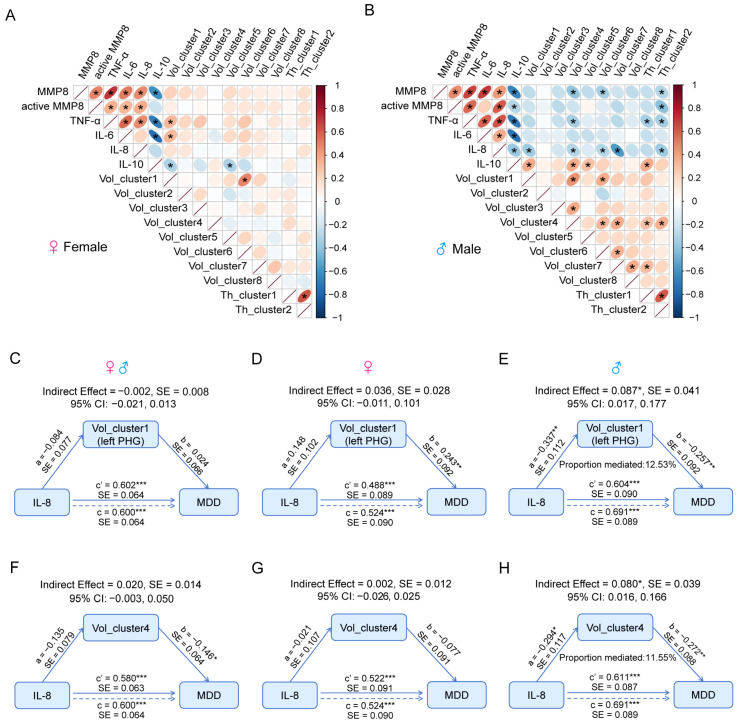
Correlations among inflammation, structural imaging phenotypes, and depression. (**A**,**B**) The correlation matrix demonstrates the associations between inflammatory factors and structural imaging phenotypes with sex-by-diagnosis interaction in females (**A**) and males (**B**) while controlling for age and BMI. The ellipses in the plot symbolize the correlation between variable pairs. Larger and more elongated ellipses denote stronger correlations, while smaller or circular ones suggest weaker or no correlations. Red, upward-pointing ellipses indicate positive correlations, whereas blue, downward-pointing ones represent negative correlations. Statistically significant correlations are marked with asterisks (*p* < 0.05). (**C**–**E**) Examining the mediating role of Vol_cluster1 in the association between IL-8 and depression across all participants (**C**), as well as separately for females (**D**) and males (**E**). (**F**–**H**) Investigating the mediating role of Vol_cluster4 in the relationship between IL-8 and depression among all participants (**F**), as well as individually for females (**G**) and males (**H**). Note: vol, volume; th, thickness. * *p* < 0.05, ** *p* < 0.01, *** *p* < 0.001.

To investigate the potential impact of elevated inflammation on sex-related brain structure in MDD, correlation analyses were conducted between the plasma concentrations of inflammatory factors and the 10 structural metrics that exhibited a sex-by-diagnosis interaction. In females, positive correlations or trends were observed between pro-inflammatory markers and these brain structures, while anti-inflammatory IL-10 exhibited a negative correlation (Figure 6A). Conversely, in males, pro-inflammatory factors displayed pronounced negative associations with the 10 structural phenotypes, while demonstrating positive correlations between these structural phenotypes and IL-10 (Figure 6B). The findings suggest that inflammation may exert divergent effects on sex-related imaging phenotypes in women and men with MDD, particularly demonstrating a pronounced negative association between elevated levels of inflammation and these structural metrics in men.

Finally, mediation analyses were conducted to examine whether the sex-differentiated brain phenotypes in MDD mediate the relationship between IL-8 and depression, considering that IL-8 exhibits a sex-by-diagnosis interaction and a significant association with depressive syndromes. Additionally, we explored potential differences in this mediation across sexes. The results indicate that in males exclusively, Vol_cluster1 (left PHG) significantly mediated the relationship between IL-8 and depression (indirect effect = 0.087, ***p* = 0.035**; Figure 6E), as well as Vol_cluster4 (areas in upper part of left precentral and postcentral gyri) (indirect effect = 0.080, ***p* = 0.041**; Figure 6H). However, no significant mediation effects were observed for all participants (Figure 6C,F) or females (Figure 6D,G). Considering the replication of PHG in the sex-by-diagnosis interaction on GMV in the independent dataset, these findings suggest that higher levels of peripheral IL-8 may influence depression indirectly through their effect on PHG in males.

## 3. Discussion

In this study, we conducted a pioneering multimodal study to explore the interplay between inflammation and brain structural phenotypes in the context of sex disparities in MDD. The findings reveal significant interactions between sex and MDD in relation to brain morphology, with post hoc analyses indicating pronounced alterations exclusively in male patients. Furthermore, the MDD group exhibits an elevated level of inflammation that has been found to have divergent effects on these sex-differentiated brain phenotypes in MDD, particularly demonstrating significant negative correlations among males. Among the examined inflammatory factors, IL-8 emerges as a prominent marker with sex-by-diagnosis interaction, which displays a larger effect size in males. Interestingly, mediation analyses suggest elevated peripheral IL-8 levels may indirectly contribute to depression in males by affecting the volume of PHG.

Our study extends current knowledge by delineating the effects of MDD on brain multimodal structure and microstructure, particularly in regions such as the prefrontal, limbic, insular, posterior cingulate cortices, and precuneus, which are well-established in the depression literature. For example, our findings highlight similar brain regions to a recent meta-analysis, which presents compelling evidence of neuronal metabolism and integrity alterations, specifically reductions in *N*-acetyl aspartate (NAA) among individuals with MDD [22]. This suggests the significant role of metabolic impairment in the neurobiological alterations and pathophysiology of depression. Additionally, both cortical thickness and GM diffusion metrics demonstrate abnormalities in the frontoparietal operculum, a key region for linguistic abilities, sensory perception, emotional response, and cognitive processing, with the diffusion metrics especially pointing to issues throughout the right operculum, including frontoparietal and temporal components [23]. The increase in GM MD suggests an expansion of the interstitial space, potentially resulting from a decrease in neural or glial cell density or size or a reduction in the water exchange rate between intra- and extracellular compartments [24]. Furthermore, the post-mortem DTI and histopathology findings also indicate that the increase in neuron density could potentially result in an elevation of FA in demyelinated GM lesions [25]. Therefore, the observed increase in MD and decrease in FA suggests reduced neural or glial cell density or size in the MDD group, which may also underlie the observed decrease in cortical thickness.

In contrast to the main MDD effect, the significant sex-by-diagnosis interactions were mainly located in the limbic structures (such as PHG, ACC, NAc, and olfactory cortex), frontoparietal operculum, and supramarginal and temporal regions. These brain regions are implicated in cognition, memory, emotion regulation, and somatosensory perception [26]. A study using positron emission tomography (PET) found that medication-free women with MDD were found to have higher normalized serotonin synthesis in multiple regions involved in emotion regulation, including the inferior frontal gyrus and limbic system, such as ACC, PHG, precuneus, superior parietal lobule, and lingual gyrus compared to men with MDD [27]. This finding may account for the increased GM volume or cortical thickness observed in female patients in these identified brain regions. Moreover, we identified similar brain regions to a prior study that reported sex-specific alterations in GM volume in the orbitofrontal, olfactory, and calcarine cortices, along with sex-specific differences in the right PHG and right calcarine cortex responses to the task and “Go/No-Go” condition [28]. Another fMRI finding revealed that depression is characterized by distinct abnormalities in functional connectivity within the default mode network (DMN) with significant sex-specific differences; notably, hyperconnectivity in this network is primarily driven by men, while various connections exhibit altered patterns in opposing directions between men and women with depression [29]. Sex-specific and suicide-related neurochemical alterations in the kynurenine pathway of the ACC have been detected in the postmortem brain tissue of individuals with MDD, with a decrease found in female MDD subjects for kynurenic acid (KYNA) levels and the KYNA/quinolinic acid (QUIN) ratio [30].

The interplay between the peripheral immune system and CNS is a key pathophysiological feature in depression [19]. Neuroinflammation, triggered by astrocyte damage, increases BBB permeability and facilitates the recruitment of peripheral monocytes by activated microglia (immune cells of the brain) and the subsequent stimulation of cytokine production, thereby exacerbating inflammation [31]. Microglial cells can reshape neural structures, regulate synaptic transmission, and influence behavior [32]. In our study, we observed that elevated inflammation levels may have divergent effects on sex-differentiated brain structural phenotypes in MDD, with men showing a general negative correlation. This finding could be attributed to significant differences in the activation states of microglial cells between males and females with depression [33]. In male rats, stress increases microglial activation and dendritic retraction in mPFC, leading to deficits in prefrontally mediated behaviors. In contrast, females exhibit stress-induced dendritic proliferation in mPFC and enhancements in prefrontally mediated behaviors [34]. Recent evidence on the differential transcriptome in postmortem brain tissues across several limbic and frontal cortices have demonstrated notably opposite transcriptional alterations between the sexes and revealed that gene expression changes in male patients with MDD indicate a reduction in synaptic markers and an increase in microglia and inflammation markers, while these gene expression changes in female patients suggest inverse alterations [35,36]. At the cellular level, sex-specific analysis of transcriptomic changes using single-nucleus RNA sequencing in dlPFC also revealed that males primarily exhibited decreased alterations in deep-layer excitatory neurons, astrocytes, and oligodendrocyte precursor cells, while females showed predominant changes in microglia, with pro-inflammatory and anti-inflammatory pathways simultaneously downregulated [37]. The above evidence also explains the molecular and cellular mechanisms behind significant reductions in GMV and cortical thickness in brain regions with sex-by-MDD interaction in male patients, while the opposite changes observed in female patients may be due to increased neurites and synapses.

Inherent differences in the immune response between the sexes may also influence the relationship between immunity and MDD [19]. While women are often considered to have a greater vulnerability to autoimmune diseases and mood disorders, some evidence has indicated that men with depression exhibit higher levels of circulating inflammation compared to their female counterparts [38]. Specifically, IL-6 serum levels indicate a sex interaction, with men exhibiting a stronger association between IL-6 and depressed mood [39]. A population-based study also linked elevated CRP levels to higher depressive symptoms in men, independent of other risk factors, whereas obesity confounded the relationship in women [40]. In our study, a significant sex-by-diagnosis interaction was observed for IL-8, with male patients exhibiting higher IL-8 level compared with females. A sex-specific role for IL-8 has been found in predicting treatment response in MDD; lower baseline IL-8 levels and an increase during treatment correlate with better outcomes, especially in females [6,41]. Furthermore, higher IL-8 levels may be protective against suicide risk in females with mood and anxiety disorders and enhanced cognitive function in depressive patients [42]. However, the contradiction between high levels of IL-8 in MDD and this neuroprotective effect may primarily apply to cases characterized by low concentrations of pathological biomarkers [43]. Conversely, a study found elevated IL-8 levels in the cerebrospinal fluid of depressive patients, particularly two male patients with chronic or recurrent severe depression and a history of suicide attempts [44]. Although research on IL-8’s sex-specific role in MDD is not uniformly conclusive, it suggests that IL-8 may play a significant role in the pathogenesis or maintenance of depression in certain sex or diagnostic subsets of patients rather than in all individuals with MDD [19]. Furthermore, IL-8 may influence depression indirectly in males only through their effect on the volume of PHG, a brain region involved in visuospatial processing, episodic memory, emotional processing, and contextual associative processing [45]. Its potential role as a sex-specific region warrants further investigation.

Our findings indicate that male patients experience more pronounced brain damage and higher IL-8, even though female patients score significantly higher on the depressive scale compared to male patients. Traditionally, MDD is perceived as being female dominated, potentially due to the fact that women often display more prominent and characteristic symptoms of depression and seek medical consultations at higher rates than men, resulting in an overrepresentation of women among individuals with MDD [46]. However, the suicide rates among males are three times higher than those among females [47]. It is also important to note that the majority of research on the pathogenesis of depression, often derived from preclinical animal studies or fundamental experiments, has historically focused on male rodents or neglected to consider sex as a variable [5,48]. This has contributed to the tendency to associate potential pathologies and pathogenic mechanisms with the concept of epidemiologically female-dominated depression, thereby overlooking the significant role that the severity of depression in males plays in these mechanisms. As the evidence has shown, hyperconnectivity within the DMN, which correlated with depression status, occurred almost exclusively in men across multiple subject cohorts recruited and scanned under differing conditions [29].

As has also been found, while higher levels of inflammation (IL-8) are observed in male patients, reports of HAMD depressive severity have indicated a negative correlation. This discrepant phenomenon could stem from societal expectations that discourage men from openly expressing depressive emotions, leading to a tendency to underestimate their emotional distress in clinician evaluations [49]. Alternatively, men might employ different coping mechanisms (such as avoidance, activity, or anger) to mask their emotional discomfort [50]. As a result, they may experience emotional or behavioral symptoms that do not align with their physiological inflammation levels and are not easily recognized or assessed using traditional HAMD criteria. Efforts are being made to raise awareness of men’s depression. The recently published ICD-11 included irritability and an absence of emotional experience (emptiness) as valid components of depressive episodes [51]. These changes are seen as a substantial improvement for identifying depressive disorders in males with an externalizing phenotype, underscoring the importance of recognizing and addressing the unique manifestations of depression in males to improve mental health outcomes and reduce the incidence of suicide [52].

There are certain limitations related to this study. First, DTI is a non-invasive method for evaluating the brain microstructure; however, standard DTI models may not accurately capture the complex organization of gray matter, including glial cells, neuronal bodies, and neurites. Multi-shell diffusion models, such as neurite orientation dispersion and density imaging (NODDI) and soma and neurite density imaging (SANDI), may provide more biological insights into sex-related brain abnormalities in depression [53,54]. Second, the limited sample size and single-center data may impede the generalizability of our findings. Moreover, our sample is mainly focused on first-episode treatment-naive individuals. To enhance our understanding of the relationship between inflammation and MDD, it would be beneficial to consider factors such as the duration of illness and number of depressive episodes and include a more diverse population with varying treatment histories. Third, our current research focused on elucidating the intricate interplay between inflammation and neuroanatomical structures in the context of sexual dimorphism at a single time point. A longitudinal study design would allow for the observation of changes over time, providing a more dynamic understanding of the relationship between inflammation and brain morphology in MDD. Finally, our research lacks exploration of the underlying mechanisms. It is imperative to investigate the molecular or genetic mechanisms that underlie these sex differences in both male and female subjects, with a particular focus on elucidating the intricate interplay between neuroinflammation and peripheral inflammation.

## 4. Materials and Methods

### 4.1. Participants

Initially, 193 individuals with MDD (106 female: 87 male) were included in this research. The inclusion criteria for individuals with MDD were as follows: (1) MDD diagnosis based on the International Classification of Diseases, 10th Edition (ICD-10); (2) HAMD-17 scores exceeding 17; (3) age ranging from 18 to 40 years; and (4) either first episode of untreated MDD or no history of any psychiatric treatments within the past 6 months, including medications, psychotherapy, and neuromodulation therapy. Patients with comorbid Axis I psychiatric disorders, intellectual disability, multiple personality disorders, heart diseases, and any systemic inflammatory or autoimmune disease were excluded from the study. The initial healthy control (HC) group included 138 age-matched participants (76 females: 62 males). The inclusion criteria for an HC are as follows: (1) without mental illness coded using ICD-10, (2) age range 18–40 years, and (3) no systemic inflammatory or immune diseases. The disease-related information, including age, sex, body mass index (BMI), family history of mental disorders, self-injury behavior, suicidal ideation, suicidal attempts, childhood trauma, and history of drinking was collected. Further, the severity of depression in all participants was measured using the HAMD-17 and BDI-II. The baseline data of the samples included in this study are shown in Table 1.

All participants with substantial brain lesions and contraindications to scanning were further excluded. The final numbers included in the study were 174 individuals with MDD (94 female: 80 male) and 133 HCs (75 females: 58 males). The detailed screening process for individuals with MDD and HCs can be found in Supplementary Figures S1 and S2, respectively.

We further included 126 subjects (40 female MDD: 47 HC; 18 male MDD: 21 HC) collected from two additional datasets as the independent replication dataset (OpenNeuro: https://openneuro.org/datasets/ds003653/versions/1.0.0 (accessed on 12 April 2024) and https://openneuro.org/datasets/ds000171/versions/00001 (accessed on 12 April 2024)) [55,56]. The demographic of each site is described in Supplementary Table S1.

### 4.2. Assessment of Inflammation

A total of 85 patients with MDD (female vs. male: 48 vs. 37) and 77 HCs (female vs. male: 44 vs. 33) voluntarily provided their blood samples. The participants’ venous blood was collected in the morning after fasting for a minimum duration of 9 h and subsequently subjected to centrifugation for plasma isolation. The processes for blood sample collection and handling were standardized, with no occurrences of hemolysis; therefore, all provided blood samples underwent enzyme-linked immunosorbent assays (ELISA). The plasma concentrations of MMP8, active MMP8, pro-inflammatory cytokines tumor necrosis factor (TNF) -α, interleukin (IL) -6, IL-8, and anti-inflammatory cytokine IL-10 in samples are collected due to their significant correlations with MDD and neural plasticity, as well as their widespread utilization in research [57,58]. They were determined using a sandwich ELISA according to the manufacturer’s instructions (KYY-0106H1, KYY-0106H1, KYY-0122H1, KYY-0049H1, KYY-1558H1, and KYY-0066H1, respectively, for MMP8, active MMP8, TNF-α, IL-6, IL-8, and IL-10, Keyybio, Jinan, Shandong, China).

### 4.3. MRI Images Acquisition

The T1-weighted (T1w) structural images and diffusion-weighted images (DWI) were acquired on a 3.0T GE scanner (Discovery MR750w, GE Healthcare, Milwaukee, WI, USA) in Qilu Hospital. A high-resolution 3D T1w gradient echo sequence scan was collected per participant based on the following parameters: repetition time (TR) = 0.0085 s; echo time (TE) = 0.0032 s; matrix = 256 × 256; field of view (FOV) = 256 × 256 mm^2^; flip angle (FA) = 12°; inversion time (TI) = 0.45 s; slice thickness = 1.0 mm isotropic voxels, with no gap. The diffusion data were acquired with an axial spin-echo echo-planar imaging (SE-EPI) sequence with the following parameters: TR = 10,000 ms; TE = 100 ms; FOV = 256 × 256 mm^2^; matrix = 128 × 128; 69 slices; slice thickness = 2.0 mm isotropic voxels, with no gap. There were 2 different b-value shells (0 and 1000 s/mm^2^) and 67 diffusion encoding directions (3 and 64 per b-value shell) for each subject with both anterior–posterior (AP) and posterior–anterior (PA) fold-over encoding directions.

### 4.4. Structural Data Processing

We conducted VBM and SBM analyses using the Computational Anatomy Toolbox 12 (CAT12, v12.8.2, http://www.neuro.uni-jena.de/cat/ (accessed on 8 June 2023)), implemented in the Statistical Parametric Mapping analysis package (SPM12, http://www.fil.ion.ucl.ac.uk/spm/software/spm12/ (accessed on 8 June 2023)) that offers computational anatomy capabilities within MatLab 2020a. T1 image preprocessing was performed using the CAT12 VBM default setting. See Figure 1A for a concise overview of the pipeline for the structural-level metrics of GM.

The initial voxel-based processing commenced with denoising, followed by internal resampling, bias correction, affine registration, and standard SPM unified segmentation [59]. Subsequently, the refined voxel-based processing utilized the outcomes from unified segmentation for intricate steps including skull-stripping, brain parcellation, white matter hyperintensity detection, and local intensity transformation to facilitate optimal segmentation, enhanced by partial volume estimation and spatial normalization to a 1.5 mm isotropic standard space through Geodesic Shooting [60]. Finally, they were spatially smoothed with a 6 mm full-width at half-maximum (FWHM) Gaussian filter to enable statistical analysis.

Surface-based processing after voxel-based steps utilizes a projection-based thickness (PBT) for cortical thickness estimation and surface reconstruction [61]. Finally, the estimated cortical thickness values were transferred onto the Freesurfer “FsAverage” template, with spatial registration combined with spatial smoothing using a 12 mm FWHM Gaussian kernel prior to statistical analysis.

### 4.5. Diffusion Data Processing

#### 4.5.1. Preprocessing

The preprocessing and quality control of diffusion data were conducted using micapipe (v1.4.0), a processing pipeline that offers a robust framework for analyzing multimodal MRI data [62]. Briefly, for structural images, each T1w run was LPI reoriented, deobliqued, and intensity non-uniformity corrected. Subsequently, T1w images were non-linearly registered to the MNI152 standard brain template using ANTs [63]. For diffusion data, all raw DWI scans were aligned using a rigid-body registration and concatenated. Then, the concatenated DWI images underwent denoising by MP-PCA and Gibbs ring correction, as well as corrections for susceptibility-induced distortions, eddy-current-induced distortions, and motion using eddy and topup implanted in FSL (https://fsl.fmrib.ox.ac.uk/fsl/fslwiki/FSL (accessed on 8 June 2023)). A b0 image was extracted and linearly registered to an individual structural image. All original images were visually inspected to ensure high data quality, and further quality assurance was conducted by examining the registration and tissue segmentation. Next, the preprocessed DWI data was used to estimate tensor files, including mean diffusivity (MD) and fractional anisotropy (FA) to access microstructural properties. The previously obtained linear and non-linear transformation matrices were then applied to all diffusion parameter maps for conducting GBSS in standard template space. See Figure 1B for a concise overview of the pipeline for the microstructural-level metrics of GM.

#### 4.5.2. GM-Based Spatial Statistics

To compare the diffusion properties of whole-brain GM between groups, we employed GBSS to extract diffusion parameters from the core voxels within GM [64,65]. This method can effectively mitigate the influence of partial volume effects from white matter and cerebrospinal fluid. To achieve the GM skeleton, all skull-stripped T1w images were segmented to obtain probabilistic cortical tissue maps and then transformed to the standard MNI space. Then, the mean cortical map was produced by merging the aligned cortical probability maps and skeletonized to retain only the core of highly probable cortical voxels. A threshold of GM fraction >0.65 in >70% of the subjects was set to extract the skeleton of GM in this research to guarantee both the probability of GM and the integrity of the skeleton. For each subject, the cortical measurements (transformed MD and FA) were projected onto the cortical skeleton by identifying the maximally probable cortical voxels in spatially transformed cortical probability maps and keeping only data from nearby voxels with maximal cortical probability for projection onto the skeleton.

### 4.6. Validation of Sex-by-Diagnosis Interaction

To verify the findings obtained in the standard template space concerning the interaction between diagnosis and sex, we conducted region of interest (ROI) analyses by converting the significant clusters back to the individual space. We obtained the relative volume and mean thickness within each cluster identified in VBM and SBM for each individual’s raw space for further group comparisons. Subsequently, we calculated the effect size of these clusters using partial η^2^. Regarding replicability, we extracted value from the independent dataset that underwent the same preprocessing pipeline to obtain the metric derived from the masks indicating statistically significant sex-by-diagnosis interaction effects in our samples. The respective effect sizes were then computed using the same methods as described earlier.

### 4.7. Statistics

The statistical analyses of the demographic and clinical data were performed using JASP software (version 0.19.1) (https://jasp-stats.org/ (accessed on 6 April 2024)). For neuroimaging analyses, 2-factor 2-level analysis of covariance (ANCOVA) was fitted separately for cortical thickness, GM volume, MD, and FA to test for the effects of MDD diagnosis, sex, and sex-by-diagnosis interaction. All models included age and BMI as a covariate of no interest. For VBM, the modulated normalized GM segmentation map of each participant was globally scaled with the total intracranial volume (TIV) to enable group comparisons based on the relative volume. We subsequently conducted separate two-sample unpaired *t*-tests for females and males to examine the effects of MDD within each sex. All the results were adjusted for multiple comparisons at a whole-brain level using threshold-free cluster enhancement (TFCE) with 5000 random permutations to maintain a family-wise error rate (FWE) of *p* < 0.05. The statistical results at the voxel level are projected onto surfaces using Surf Ice (v2.1.70-0) (https://www.nitrc.org/projects/surfice/ (accessed on 1 December 2023)).

For the analyses of inflammatory factors and ROI-wise structural metrics, we utilized JASP software and applied Tukey’s HSD test, a robust method for post hoc pairwise comparisons following ANCOVA, to adjust for multiple comparisons. To examine the relationship between inflammatory factors and brain regions with significant sex-by-diagnosis interactions, the mean GM measurements within each cluster were extracted and correlation analyses were conducted with age and BMI controlled. For these correlational analyses, false discovery rate (FDR) correction was applied to account for multiple comparisons. Additionally, GraphPad Prism 9.5 (https://www.graphpad.com/ (accessed on 1 December 2023)) and corrplot packages in R (version 4.2.1) were utilized to plot and visualize these statistical results.

Mediation analyses were subsequently conducted using SPSSAU (https://www.spssau.com (accessed on 1 September 2024)) to estimate the role of these imaging phenotypes in mediating the relationship between specific inflammatory factors and MDD. Specifically, separate mediation analyses were performed for all participants regardless of sex, as well as for females and males, respectively, to explore potential sex differences in the mediation effects of the sex-related structural measurements in MDD. For all participants, age, BMI, and sex were included as covariates of no interest. For females/males only, age and BMI were included as covariates. The significance of mediation effects was assessed using 5000 bootstrap realizations to calculate a bootstrap 95% confidence interval (CI), where a significant indirect effect was indicated when the bootstrap 95% CI did not include zero and *p* < 0.05.

## 5. Conclusions

Our study identified sex-specific alterations in GM structure and microstructure, as well as the central effects of peripheral inflammation. The findings highlight significant GM abnormalities in males with MDD, even though higher depressive scores are observed in female patients. This discrepancy might have clinical implications, enabling an increased awareness of depression in men and encouraging them to express their mental health concerns and seek medical care. Additionally, our findings suggest an indirect influence of IL-8 on male depression, mediated through parahippocampal volume, indicating that IL-8 may serve as biomarker for specific diagnostic subgroups. These results require further exploration with larger, more diverse samples that consider illness duration. Overall, this study provides further evidence suggesting potential sex differences in MDD, highlighting the importance of considering sex as a significant variable in understanding the relationship between peripheral inflammation and brain changes and developing targeted interventions for MDD.

## Figures and Tables

**Table 1:** Demographic and clinical features.

	MDD, n = 174			HC, n = 133		
	Female, n = 94	Male, n = 80	t/χ^2^ (*p*)	Female, n = 75	Male, n = 58	t/χ^2^ (*p*)
Age (years)	24.14 ± 5.20	23.71 ± 4.56	0.67 (0.91)	24.08 ± 2.94	24.24 ± 2.84	0.22 (1.00)
BMI (kg/m^2^)	21.45 ± 3.72	23.94 ± 4.60	−4.38 (**<0.001**)	20.34 ± 3.46	22.77 ± 2.60	−3.73 (**<0.001**)
HAMD-17	28.93 ± 6.49	25.50 ± 5.52	4.46 (**<0.001**)	2.29 ± 3.00	3.28 ± 3.64	−1.12 (0.68)
BDI-II	37.16 ± 9.17	32.09 ± 10.15	4.27 (**<0.001**)	2.83 ± 3.74	4.76 ± 5.15	−1.41 (0.50)
TIV (cm^3^)	1456.19 ± 112.41	1629.09 ± 148.81	−8.9 (**<0.001**)	1479.83 ± 114.54	1649.56 ± 136.24	−7.60 (**<0.001**)
Family history of mental illness (yes/no)	13/81	9/71	0.26 (0.61)	1/74	0/58	0.78 (0.38)
Self-injury behavior (yes/no)	48/46	28/52	4.53 (**0.03**)	2/73	0/58	1.54 (0.21)
Suicidal ideation (yes/no)	80/14	70/10	0.12 (0.65)	3/72	5/53	1.24 (0.27)
Suicidal attempts (yes/no)	23/71	21/59	0.07 (0.79)	0/75	0/58	-
Childhood trauma (yes/no)	23/71	18/62	0.09 (0.76)	1/74	2/56	0.66 (0.42)
Drink (yes/no)	29/65	28/52	0.34 (0.56)	4/71	20/38	18.79 (**<0.001**)

Note: MDD, major depressive disorder; HC, healthy control; BMI, body mass index; TIV, total intracranial volume; HAMD-17, Hamilton Depression Rating Scale; BDI-II, Beck Depression Inventory-II. Descriptive statistics of continuous variables are recorded as mean ± SD.

## Data Availability

Data is contained within the article or Supplementary Material.

## Supplementary Materials

The following supporting information can be downloaded at: https://www.mdpi.com/article/10.3390/ijms252413412/s1.
