# Supplementary material for: Sex Differences in Depression: Insights from Multimodal Gray Matter Morphology and Peripheral Inflammatory Factors
Source: Int J Mol Sci. 2024 Dec 14;25(24):13412. doi: 10.3390/ijms252413412 (PMC11677592; doi:10.3390/ijms252413412)
Supplement: Supplementary file 1 [file ijms-25-13412-s001.zip › ijms-3353500-supplementary.pdf]

Supplementary Information

Sex Differences in Depression: Insights from Multimodal Gray Matter Morphology and Peripheral Inflammatory Factors

Supplementary Figure S1. Flowchart of screening process for individuals with MDD

Supplementary Figure S2. Flowchart of screening process for healthy controls

Supplementary Figure S3. Main effect of sex

Supplementary Figure S4. Replicability of sex-by-diagnosis interaction effect in the independent dataset.

Supplementary Table S1. Demographic of replication dataset

Supplementary Table S2. Sex-by-diagnosis interaction on GM volume measured by VBM

Supplementary Table S3. Sex-by-diagnosis interaction on cortical thickness measured by SBM

Supplementary Table S4. ROI-wise analyses of the sex-by-diagnosis interaction on GM volume and cortical thickness

Supplementary Table S5. ROI-wise replication of the sex-by-diagnosis interaction on GM volume in the independent dataset

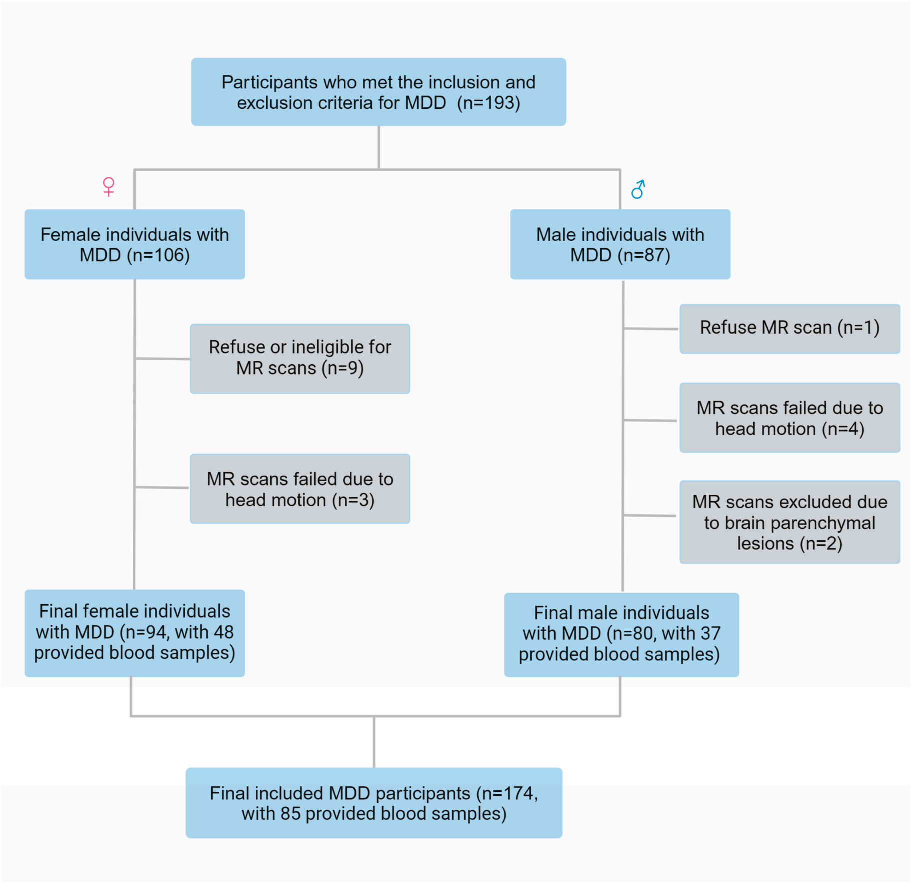

Supplementary Figure S1. Flowchart of screening process for individuals with MDD. Note: MDD, major depressive disorder; MR, magnetic resonance.

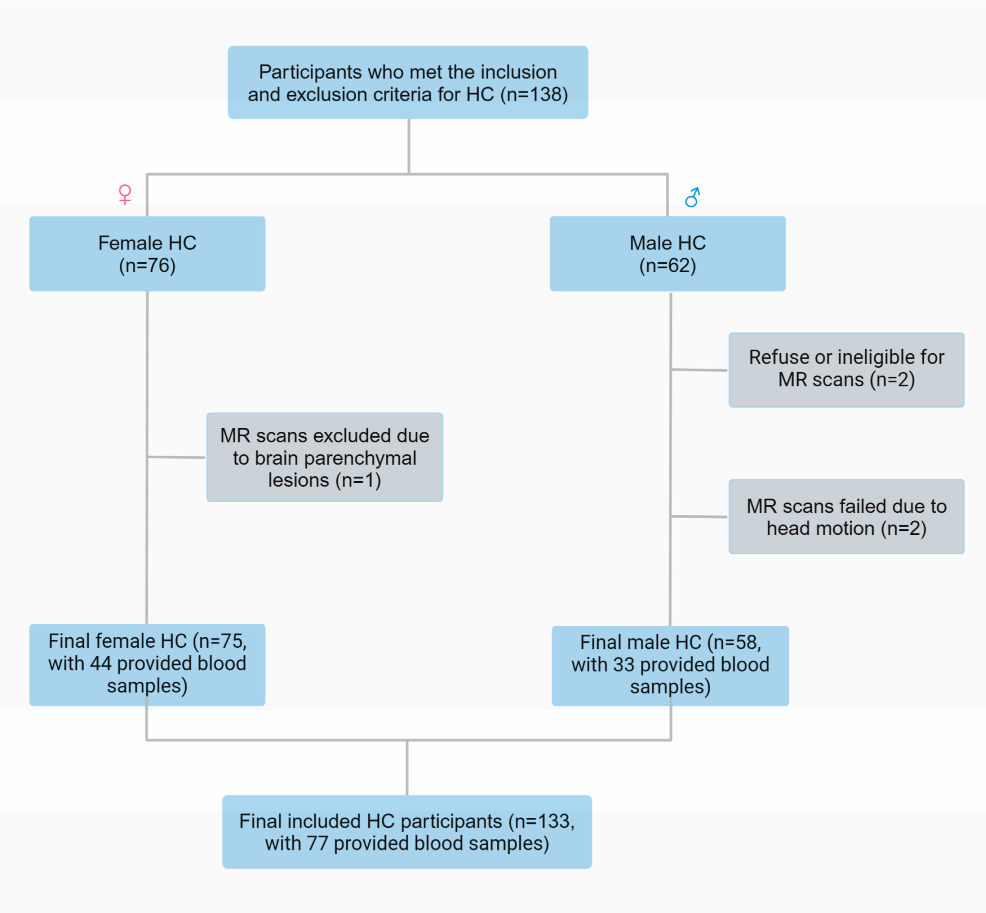

Supplementary Figure S2. Flowchart of screening process for individuals with healthy controls. Note: HC, healthy controls; MR, magnetic resonance.

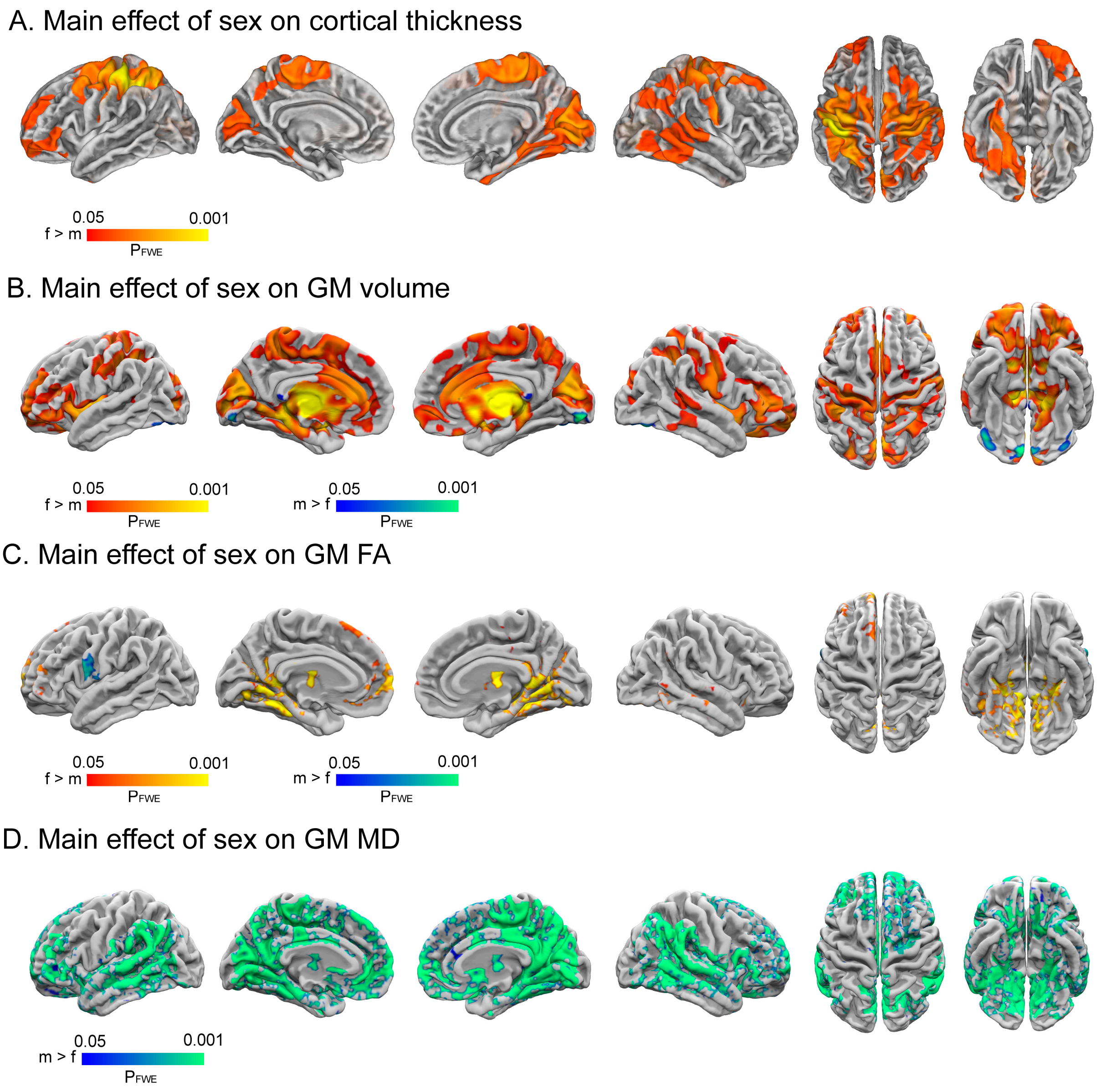

Supplementary Figure S3. The main effect of sex on cortical thickness (A), gray matter volumes (B), MD (C) and FA (D). The red-yellow areas are regions where the cortical thickness(A), GM volume (B) and FA (D) is significantly higher in females compared with males. Areas in blue-green indicate regions where the GM volume (B) MD (C) are significantly lower in female compared with males. The statistical significances are determined at $p<0.05$ after applying FWE correction for multiple comparisons following TFCE. Note: MDD, major depressive disorder; HC, healthy control; GM, gray matter; MD, mean diffusivity; FA, fractional anisotropy.

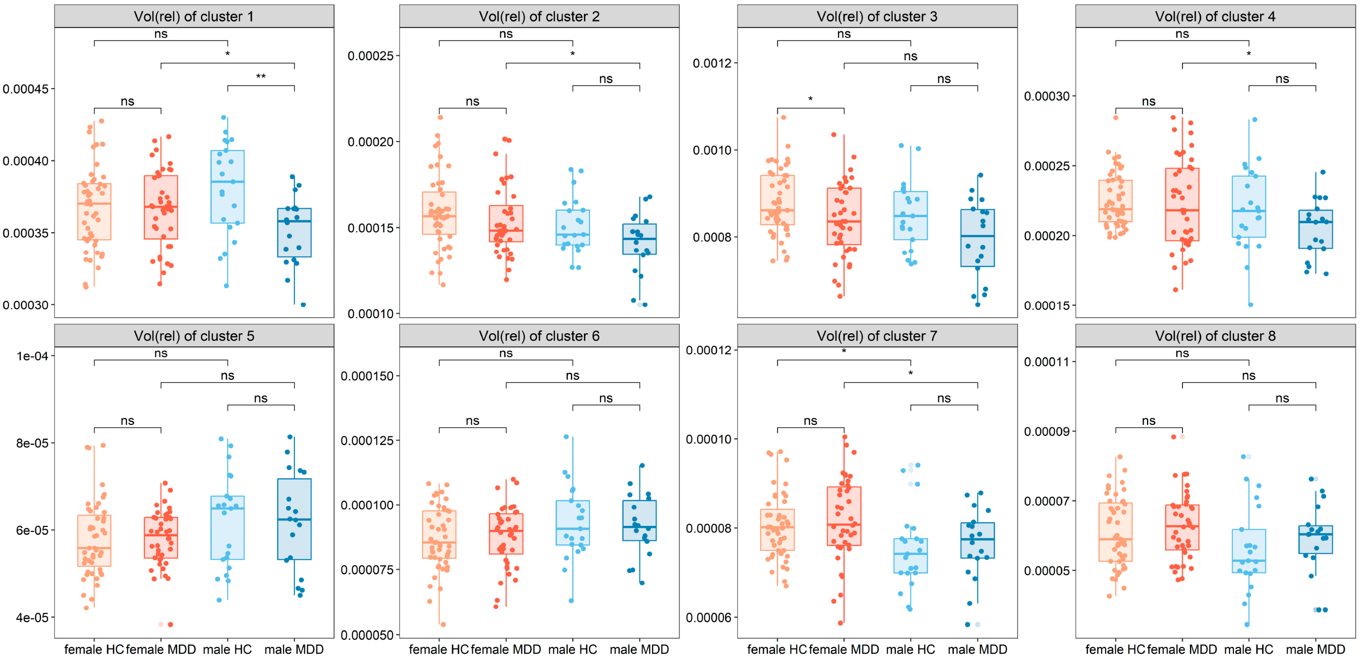

Supplementary Figure S4. Replicability of sex-by-diagnosis interaction effect in the independent dataset. The violin plots exhibit the validation of regions of interest (ROI) analysis in the independent replication dataset for each cluster determined by the interaction of sex and diagnosis on GM volume. Note: * $P < 0.05$, ** $P < 0.01$; Vol (rel): relative volume; MDD, major depressive disorder; HC, healthy control.

Supplementary Table S1. Demographic of replication dataset

	MDD			HC			All		
Site	Number	Female/Male	Age (range, years)	Number	Female/Male	Age (range, years)	Number	Female/Male	Age (range, years)
ds003653	39	29/10	28.52 (19.3-44.3)	48	36/12	29.1 (19.1-44)	87	65/22	28.78 (19.1-44.3)
ds000171	19	11/8	33.53 (18-56)	20	11/9	29/45 (18-59)	39	22/17	31.4 (18-59)

Note: MDD, major depressive disorder; HC, healthy control.

Supplementary Table S2. Sex-by-diagnosis interaction on GM volume measured by VBM

	$P_{FWE\text{-}corr}$	Cluster-Size	MNI coordinates	Overlap of AAL3 Atlas region	
Cluster 1	0.015	1507	-18, -26, -29	40%	Left Parahippocampal gyrus
				23%	Left Lingual gyrus
				6%	Left Lobule IV-V of cerebellar hemisphere
				5%	Left Hippocampus
				3%	Right Lobule IV-V of cerebellar hemisphere
				2%	Right Lobule III of cerebellar hemisphere
				2%	Lobule III of vermis
				2%	Right Lingual gyrus
				1%	Left Fusiform gyrus
Cluster 2	0.020	750	17, -6, 18	48%	Right Caudate nucleus
				8%	Right Ventral lateral
				2%	Right Ventral posterolateral
Cluster 3	0.022	3006	9, 29, -6	26%	Left Anterior cingulate cortex-pregenual
				13%	Left Anterior cingulate cortex-supracallosal
				9%	Right Anterior cingulate cortex-subgenual
				8%	Left Anterior cingulate cortex-subgenual
				7%	Right Anterior cingulate cortex-pregenual
				7%	Left Olfactory cortex
				6%	Left Nucleus accumbens
				4%	Left Caudate nucleus
				3%	Right Olfactory cortex
				1%	Left Superior frontal gyrus-medial
Cluster 4	0.027	1108	-17, -5, 68	39%	Left Superior frontal gyrus-dorsolateral
				36%	Left Precentral gyrus
				19%	Left Supplementary motor area
				2%	Left Postcentral gyrus
Cluster 5	0.031	270	-3, -86, 33	96%	Left Cuneus
Cluster 6	0.037	471	2, -60, 8	23%	Left Precuneus
				16%	Right Lingual gyrus
				12%	Right Calcarine fissure and surrounding cortex
				10%	Left Calcarine fissure and surrounding cortex
				9%	Lobule IV-V of vermis
				6%	Right Precuneus
Cluster 7	0.038	337	42, -21, 2	45%	Right Insula
				33%	Right Superior temporal gyrus
Cluster 8	0.039	341	56, -8, 18	80%	Right Rolandic operculum
				10%	Right Postcentral gyrus

Note: GM: gray matter; VBM, volume-based morphometry; FWE, family-wise error; MNI, Montreal Neurological Institute; AAL3, Automated Anatomical Labeling 3.

Supplementary Table S3. Sex-by-diagnosis interaction on cortical thickness measured by SBM

	$P_{FWE\text{-}corr}$	Cluster-Size	MNI coordinates	Overlap of DK40 Atlas region	
Cluster 1	0.034	2142	53, -27, 40	34%	supramarginal
				27%	postcentral
				17%	precentral
				11%	pars opercularis
				8%	caudalmiddlefrontal
				2%	superiortemporal
				1%	rostralmiddlefrontal
Cluster 2	0.041	513	58, -17, -24	52%	middletemporal
				25%	inferiortemporal
				23%	superiortemporal

Note: SBM, surface-based morphometry; FWE, family-wise error; MNI, Montreal Neurological Institute; DK40, Desikan-Killiany 40.

Supplementary Table S4. ROI-wise analyses of the sex-by-diagnosis interaction on GM volume and cortical thickness

Measurement	ROI	Main effect of MDD		Main effect of sex		Sex-by-diagnosis interaction	
		η^2_p	F (p)	η^2_p	F (p)	η^2_p	F (p)
GM volume	Cluster 1	0.001	0.32 (0.572)	0.02	***12.42 (<0.001)	0.062	***19.88 (< .001)
	Cluster 2	<0.001	<0.01 (0.988)	0.004	1.32 (0.252)	0.036	**11.11 (<0.001)
	Cluster 3	0.004	1.08 (0.299)	0.013	*3.95 (0.048)	0.034	**10.47 (0.001)
	Cluster 4	0.037	***11.61 (<0.001)	0.002	0.48 (0.487)	0.022	**6.65 (0.010)
	Cluster 5	<0.001	<0.01 (0.946)	0.045	***14.13 (<0.001)	0.017	*5.15 (0.024)
	Cluster 6	0.002	0.53 (0.468)	0.017	*5.12 (0.024)	0.029	**8.92 (0.003)
	Cluster 7	0.010	3.18 (0.076)	0.006	1.70 (0.194)	0.033	**10.36 (0.001)
	Cluster 8	0.006	1.94 (0.165)	0.011	3.23 (0.073)	0.032	**9.96 (0.002)
Cortical thickness	Cluster 1	0.037	***11.69(<0.001)	0.006	1.84 (0.179)	0.048	***15.05 (< .001)
	Cluster 2	0.018	5.78 (0.020)	0.012	3.81 (0.052)	0.043	***13.49 (< .001)

Note: ROI, region of interest; GM, gray matter; η^2_p, partial η^2 (effect size of ANOVA).

Supplementary Table S5. ROI-wise replication of the sex-by-diagnosis interaction on GM volume in the independent dataset

Measurement	ROI	Main effect of MDD		Main effect of sex		Sex-by-diagnosis interaction	
		η^2_p	F (p)	η^2_p	F (p)	η^2_p	F (p)
GM volume	Cluster 1	0.059	**7.70 (0.006)	<0.001	0.015 (0.90)	0.051	*6.49 (0.012)
	Cluster 2	0.034	*4.24 (0.042)	0.057	**7.33 (0.008)	0.004	0.52 (0.473)
	Cluster 3	0.069	**8.97 (0.003)	0.043	*5.50 (0.021)	0.003	0.33 (0.569)
	Cluster 4	0.021	2.58 (0.111)	0.047	*6.08 (0.015)	0.01	1.18 (0.279)
	Cluster 5	<0.001	<0.01 (0.99)	0.04	*5.12 (0.025)	<0.001	<0.01 (0.96)
	Cluster 6	<0.001	<0.01 (0.97)	0.036	*4.51 (0.036)	0.002	0.19 (0.664)
	Cluster 7	0.005	0.65 (0.422)	0.084	**11.13 (0.001)	<0.001	<0.01 (0.99)
	Cluster 8	0.012	1.51 (0.220)	0.033	*4.14 (0.044)	<0.001	0.07 (0.793)

Note: ROI, region of interest; GM, gray matter; η^2_p, partial η^2 (effect size of ANOVA).
